# 8-Oxoguanine DNA Glycosylase (OGG1) Cys326 Variant: Increased Risk for Worse Outcome of Patients with Locally Advanced Rectal Cancer after Multimodal Therapy

**DOI:** 10.3390/cancers13112805

**Published:** 2021-06-04

**Authors:** Martin Leu, Theresa Riebeling, Leif Hendrik Dröge, Laura Hubert, Manuel Guhlich, Hendrik Andreas Wolff, Jürgen Brockmöller, Jochen Gaedcke, Stefan Rieken, Markus Anton Schirmer

**Affiliations:** 1 Clinic of Radiotherapy and Radiation Oncology, University Medical Center Göttingen, Robert-Koch-Str. 40, 37075 Göttingen, Germany; martin.leu@med.uni-goettingen.de (M.L.); theresa.riebeling@uksh.de (T.R.); Hendrik.droege@med.uni-goettingen.de (L.H.D.); laura.hubert@outlook.de (L.H.); manuel.guhlich@med.uni-goettingen.de (M.G.); h.wolff@strahlentherapie-muenchen.eu (H.A.W.); Stefan.rieken@med.uni-goettingen.de (S.R.); 2 Department of Nephrology and Hypertension, University Hospital Schleswig-Holstein, 24105 Kiel, Germany; 3 Medical Center, Department of Radiation Oncology, University of Regensburg, 93053 Regensburg, Germany; 4 Institute of Clinical Pharmacology, University Medical Center Göttingen, 37075 Göttingen, Germany; jbrockm@gwdg.de; 5 Clinic of General, Visceral, and Pediatric Surgery, University Medical Center Göttingen, 37075 Göttingen, Germany; j.gaedcke@gmail.com

**Keywords:** rectal cancer, neoadjuvant radiochemotherapy, reactive oxygen species, ROS, base excision repair, BER, 8-oxoguanine DNA glycosylase, OGG1, Ser326Cys, rs1052133, biomarker, germline polymorphisms, cancer-specific survival, CSS, overall survival, OS

## Abstract

**Simple Summary:**

In Western countries, the lifetime risk for rectal cancer is around 1.5%. Most patients are diagnosed with locally advanced stages. For these patients, multimodal treatment comprising radiotherapy, chemotherapy, and surgery has become the standard of care. Whereas excellent local control is achieved, still about one out of three dies from this disease. Cytotoxicity of radiochemotherapy substantially involves reactive oxygen species (ROS). In ROS-related genes, we selected eight inherited variants, for which the literature reports functional or medical effects and which occur frequently in the general population. These variants were assessed whether they impact the clinical outcome of patients with rectal cancer. We found that the OGG1 Cys326 variant, which affected 37% of the 287 patients in the sample, was strongly linked with a worse outcome, in particular cancer-specific survival. Screening for this variant may identify a particular risk subgroup of patients who may be considered for more intensified therapy and aftercare.

**Abstract:**

Despite excellent loco-regional control by multimodal treatment of locally advanced rectal cancer, a substantial portion of patients succumb to this disease. As many treatment effects are mediated via reactive oxygen species (ROS), we evaluated the effect of single nucleotide polymorphisms (SNPs) in ROS-related genes on clinical outcome. Based on the literature, eight SNPs in seven ROS-related genes were assayed. Eligible patients (*n* = 287) diagnosed with UICC stage II/III rectal cancer were treated multimodally starting with neoadjuvant radiochemotherapy (N-RCT) according to the clinical trial protocols of CAO/ARO/AIO-94, CAO/ARO/AIO-04, TransValid-A, and TransValid-B. The median follow-up was 64.4 months. The Ser326Cys polymorphism in the human OGG1 gene affected clinical outcome, in particular cancer-specific survival (CSS). This effect was comparable in extent to the ypN status, an already established strong prognosticator for patient outcome. Homozygous and heterozygous carriers of the Cys326 variant (*n* = 105) encountered a significantly worse CSS (*p* = 0.0004 according to the log-rank test, *p* = 0.01 upon multiple testing adjustment). Cox regression elicited a hazard ratio for CSS of 3.64 (95% confidence interval 1.70–7.78) for patients harboring the Cys326 allele. In a multivariable analysis, the effect of Cys326 on CSS was preserved. We propose the genetic polymorphism Ser326Cys as a promising biomarker for outcome in rectal cancer.

## 1. Introduction

In Western countries, the lifetime incidence for rectal cancer is around 1.5% of the total population. In 60%, this disease is diagnosed as UICC/AJCC (Union Internationale Contre le Cancer/American Joint Committee on Cancer) stages II or III [1]. Neo-adjuvant radiotherapy or radiochemotherapy (N-RCT) with subsequent total mesorectal excision (TME) has become the standard treatment for resectable cancer of the middle or lower third of the rectum [2,3,4,5,6]. This strategy results in improved loco-regional tumor control with excellent local relapse rates of 7% in a ten-year follow-up [7]. However, still one third of the affected individuals dies from this disease, mostly due to distant metastasis. Attempts to reduce this rate are ongoing. Intensification of the chemotherapy component prior to surgery may increase disease-free survival, with some improvements seen recently advocating for complete neoadjuvant radio- and chemotherapy [8].

To pave the way for strategies to increase the cure rate, better knowledge about the factors driving the prognosis is indispensable. Evidence for residual viable tumor cells in lymph nodes of the surgical specimen following N-RCT (i.e., ypN+) and the tumor regression grade (TRG) of the primarius are histo-pathological predictors in the long run [9].

Other approaches employ genetic features for prognosis prediction of rectal cancer upon N-RCT. Besides tumor-specific mutations, inherited germline polymorphisms are increasingly considered as biomarkers in colorectal cancer therapy, not only in terms of toxicity but also in relation to outcome. In this regard, particular attention is spent on the genes involved in detoxification of reactive oxygen species (ROS), DNA repair, or metabolism of the concomitant chemotherapy. Genotypes associated with higher ROS exposure, be it due to increased ROS formation or decreased degradation, might confer a better outcome of radiochemotherapy [10,11,12].

The working hypothesis of this study was that common germ-line polymorphisms in the genes related to ROS may affect the outcome in rectal cancer upon multimodal treatment. We report data on eight germ-line polymorphisms with a minor allele frequency >10% in Caucasians in seven genes related to ROS, with respect to patient outcome. These polymorphisms refer to genes related to ROS formation (NAD[P]H oxidase), processing (superoxide dismutases [SOD2, SOD3], myeloperoxidase [MPO], and detoxification (catalase [CAT], glutathione peroxidase 1 [GPX1]), as well as involved in removal of ROS-induced DNA damage (OGG1). We only included polymorphisms with known functional relevance based on the literature (see Table 1, in the Patients and Methods section). We found carriers of the OGG1 Cys326 allele are at risk of a significantly worse outcome, in particular concerning cancer-specific survival.

## 2. Results

### 2.1. Eligible Patients

**Figure 1:**
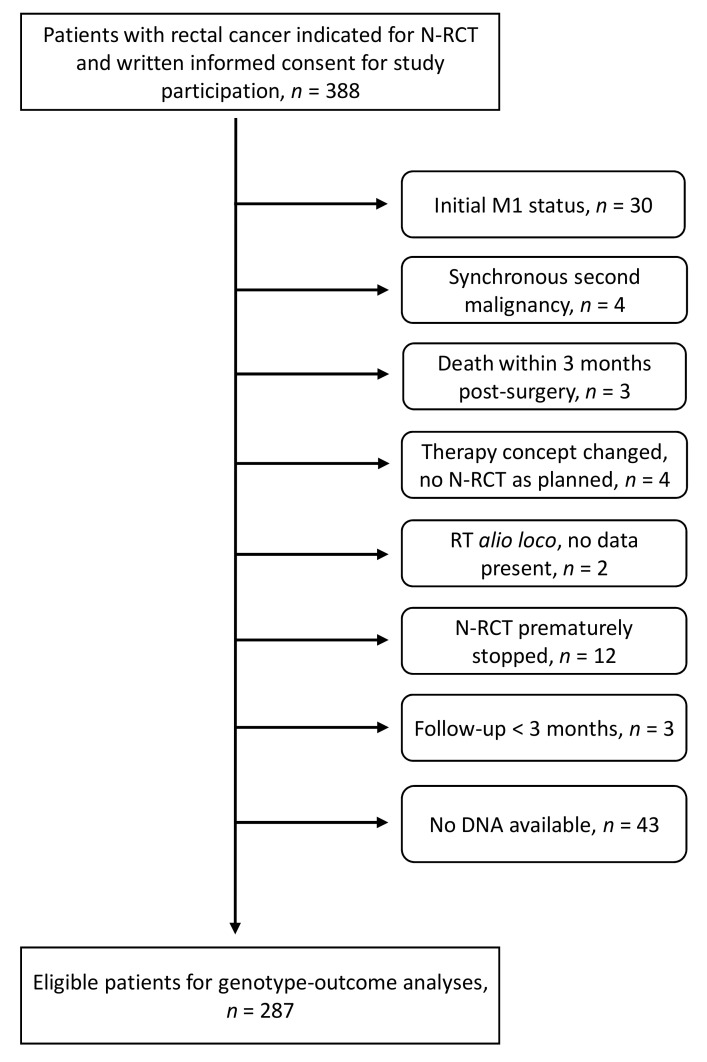
Flowchart to inform about the sample selection criteria, reducing the initial number of 388 patients to a final 287 eligible for assessment of genotypes in relation to clinical outcome. The few patients who died within three months upon surgery or those with a follow-up shorter than three months after surgery were excluded since sustainable effects of N-RCT could not be addressed in these cases.

Out of the initial dataset of 388 patients, 287 were finally eligible upon applying the exclusion criteria, as delineated in the flowchart (Figure 1) and described in the Patients and Methods section.

### 2.2. Patient Baseline, Tumor, and Treatment Characteristics in Relation to Clinical Outcome

Baseline and disease characteristics of the 287 eligible patients are summarized in Table 2. Median follow-up was 64.4 months.

**Figure 2:**
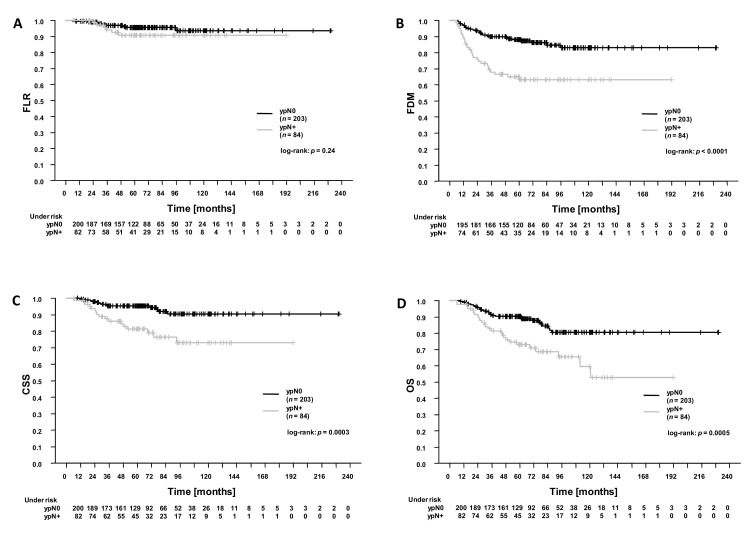
Kaplan–Meier plots for freedom of local recurrence (FLR), (**A**), freedom from distant metastasis (FDM), (**B**), cancer-specific survival (CSS), (**C**), and overall survival (OS), (**D**), depending on the histopathological nodal status (ypN) at time of surgical tumor resection upon neoadjuvant radiochemotherapy. The two groups, i.e., ypN+ vs. ypN0, were statistically compared by the log-rank test.

Baseline patient, disease, and treatment characteristics were interrogated for impact on clinical outcome. Patients with a pathologically complete response (pCR) upon N-RCT, as ascertained at tumor resection, exhibited improved outcome: OS and freedom from distant metastasis (FDM) were increased (both *p* < 0.05) and, in a borderline manner, CSS was also better (*p* = 0.07). In contrast, freedom from local recurrence (FLR) was not affected by pCR. As expected, strong associations were observed for the nodal status at time of surgical resection: viable tumor cells still were found in the lymph nodes following N-RCT (i.e., ypN+), with markedly worsened FDM, CSS, and OS (Figure 2). For instance, the risk for tumor-specific death was 3-fold higher in case of ypN+ (see Table 3, univariable Cox regression).

Since the time span of included patients extends over a long period, from 01/1998 to 08/2016, we tested whether time of treatment might have biased the outcome. Taking the date of surgery as a continuous variable revealed no impact on either outcome parameter tested (all *p* > 0.3 by univariable Cox regression analyses). Splitting the entire patient cohort at the median surgery date in two groups revealed also no relation with any of the outcome measurements (all *p* > 0.3 by log-rank test). Thus, treatment time does not convey a relevant bias in the here presented patient cohort. Over time, irradiation has technically evolved. Whereas in the earlier times three-dimensional radiotherapy (3D-RT) application was the standard, which affected 57% of our patient cohort, later on it was replaced by volumetric-modulated arc therapy (VMAT, a further advance of intensity-modulated radiotherapy where the gantry rotates around the patient, substantially reducing irradiation time). Whereas VMAT slightly improved freedom from local recurrence in comparison to 3D-RT (*p* = 0.04 by log-rank test, *p* = 0.06 by Cox regression, see Table 3), the two different techniques did not relevantly modify the other outcome parameters (all *p* > 0.2).

### 2.3. Genetic Polymorphisms and Initial Tumor Staging Parameters

The two SNPs in catalase, an enzyme for detoxifying hydrogen peroxide into oxygen and water, revealed an association with initial tumor size: hetero- or homozygous *T* variant allele carriers at rs1001179 experienced a risk ratio (RR) of 2.59 (95% confidence interval 1.17–5.71, *p* = 0.017 by Fisher’s exact test) for having initial T4 tumors versus T2–T3 in comparison to individuals with the *CC* wildtype at this site. In contrast, in case of the rs769214 *C* variant allele, the T4 tumors were less frequent than T2–T3, with a RR of 0.41 (0.18–0.92, *p* = 0.031) compared with the *TT* wildtype at this position. In terms of the initial N stage, the *T* variant at MPO rs2333227 was slightly under-represented in N+ cases with a RR of 0.87 (0.76–1.01, *p* = 0.047). More advanced N stages at diagnosis, i.e., N2-N3 vs. N0-N1, were observed for homozygous carriers of *GG* at OGG1 rs1052133 and *CC* at CAT rs769214, with RRs of 4.22 (1.39–12.82, *p* = 0.04) and 3.17 (1.26–7.94, *p* = 0.02) referred to the *GC* + *CC* and *CT* + *TT* genotypes, respectively. One should keep in mind the low numbers of the N2-N3 stages (see Table 2). Only the effect of the variant *T* allele SNP in MPO also translated into discrimination of the UICC category III vs. II in a borderline manner (RR 0.87, 0.76–1.01, *p* = 0.05). Statistical significance could not be reached for any of these features upon adjustment for multiple testing.

### 2.4. Genetic Polymorphisms and Tumor Response

Neither pathological complete response, which was achieved in 48 (17%) patients, nor ypT0 vs. ypT1-4 was dependent on any of the eight considered genetic polymorphisms. Viable tumor cells in lymph nodes upon neoadjuvant treatment (i.e., ypN+) were less likely present in carriers of the homozygous variant CC genotype of SOD2 rs4880 in comparison to the combined group of *TT* and *TC* (RR 0.56, 0.33–0.95, *p* = 0.02, recessive allele effect). A trend towards a higher rate of ypN+ was observed for patients harboring at least one *G* variant allele of OGG1 rs1052133 (RR 1.40, 0.98–2.00, *p* = 0.08, dominant allele effect). Again, statistical significance is not retained when adjusting for multiple tests.

### 2.5. Genetic Polymorphisms and Clinical Outcome

**Figure 3:**
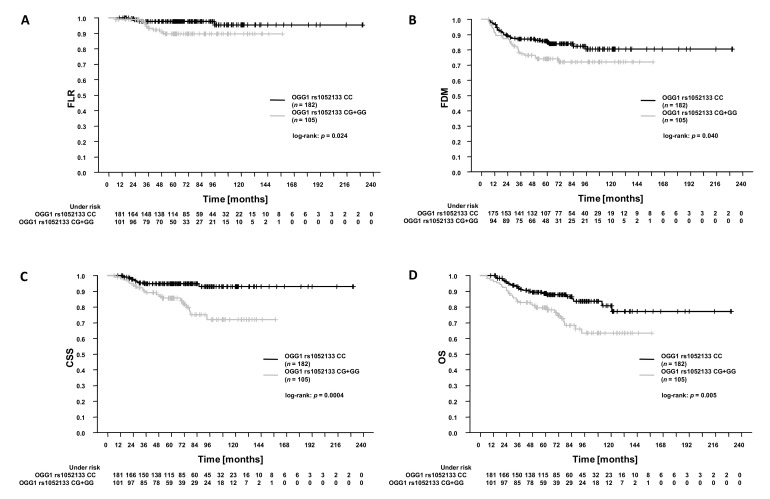
Kaplan-Meier plots for FLR (**A**), FDM (**B**), CSS (**C**), and OS (**D**), depending on the OGG1 rs1052133 polymorphism. Differences by genotype were statistically assessed by the log-rank test.

Of the eight considered genetic polymorphisms, a consistent impact on all four outcome parameters was seen for the Cys326 variant at rs1052133 of 8-oxoguanine DNA glycosylase (OGG1). The strongest association observed was with regard to cancer-specific survival (CSS) for which carriers of Cys326 exhibited a more than 3-fold increased risk. A dominant mode of action is assumed as this effect was seen when contrasting the heterozygous and homozygous Cys326 variant allele against the homozygous Ser326 wildtype allele carriers. Kaplan–Meier plots visualize these effects (Figure 3). Due to the exploratory fashion by which the genetic data were evaluated with respect to outcome, adjustment of the threshold for statistical significance was done according to the Bonferroni method. With eight genetic markers and four outcome parameters, the raw *p* values (p_raw_) are accordingly multiplied by 32 to define the threshold for the adjusted *p* values (p_adj_). In case of the association of rs1052133 with CSS, the P_adj_ was 0.02. Thus, the level of statistical significance here was still met upon correction for multiplicity testing. This approach for multiplicity testing adjustment is deemed conservative as the outcome parameters are partially linked and do not convey completely independent information. The other seven genetic polymorphisms did not demonstrate any impact on the investigated outcome.

### 2.6. Multiparametric Assessment for Clinical Outcome

The effect of rs1052133 on clinical outcome was then analyzed in a multivariable way together with age, pCR, and ypN+, which elicited a relation with outcome at *p* < 0.05 in the univariable analysis (Table 3). In this multivariable Cox regression analysis (Table 4), the strong impact of rs1052133 on outcome was retained. It turned out as the strongest predictor for CSS and OS among the investigated variables. Specifically, carriers of at least one Cys326 allele encountered a risk for a CSS event with a hazard ratio of 3.08 (95% CI 1.46–6.48, *p* = 0.003) compared to patients homozygous for the Ser326 allele adjusted for the effects of pCR and ypN+ in this multivariable analysis. Actually, the three features rs1052133, pCR, and ypN+ elicited an impact on clinical outcome largely independent from one another. With regard to OS, the association of the OGG1 polymorphism was comparable to that of pCR when considering the *p* values. Even for FLR, this genetic marker elicited a stronger impact than pCR or ypN+, albeit not reaching *p* < 0.05. For FDM, the ypN+ status was by far the strongest prognosticator in this multivariable assessment, as it was in the univariable analysis.

**Figure 4:**
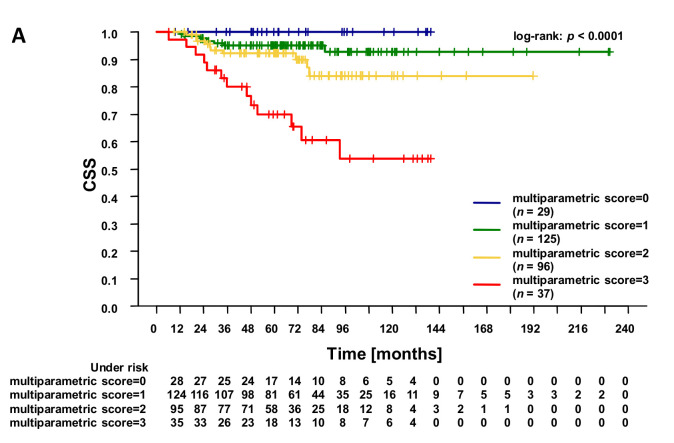

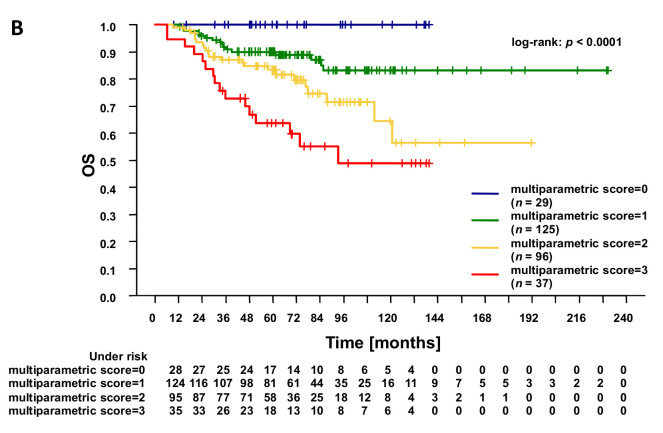
Kaplan–Meier plots for CSS (**A**) and OS (**B**), depending on a three parameter score consisting of the status of pCR, ypN, and OGG1 rs1052133. A multiparametric score of “0” was assigned if pCR was achieved, together with ypN0 and homozygosity for the wildtype allele at rs1052133. A score of “3” means that there was no pCR in combination with ypN+ and presence of the variant allele (either homo- or heterozygous). Accordingly, intermediate scores of “1” and “2” were derived for other combinations of the three considered parameters. Statistical assessment was performed by the log-rank test.

A combined multiparametric score consisting of the two histopathological features, pCR and ypN status, as well as the OGG1 rs1052133 polymorphism was generated. To each of these three parameters, values of either “0” or “1” were assigned. For instance, if pCR was achieved, it was scored as “0”, as it was in case of ypN0 and the wildtype at rs1052133. No pCR, a ypN+ status, and the presence of the variant allele at rs1052133 were rated as “1”. By adding the values for the three considered items, a multiparametric score was derived that ranged between “0” and “3”. Using this score, a strikingly strong differentiation of CSS and OS was achieved (Figure 4). Notably, none of the patients with a multiparametric score of “0” did encounter a CSS or OS event. In contrast, only about 50% of the patients with a score of “3” were free of CSS or OS events after eight years.

### 2.7. Subgroup Analyses for Parameters Affecting Clinical Outcome

When stratified by the sort of chemotherapy, which came concomitant with neoadjuvant radiotherapy, the detrimental effect of Cys326 on CSS was prominent in those patients who received 5-fluorouracil as a single cytostatic agent (*p* = 0.0002 by log-rank test). In patients given additional oxaliplatin, the impact of Cys326 was not statistically significant (*p* = 0.3, see Figure S1 in Supplementary File online). In contrast, the effect of Cys326 on CSS was similar whether adjuvant chemotherapy was given or not (see Figure S2 in Supplementary File). Regarding the ypN status upon surgery, the impact of the Cys326 variant on CSS was more pronounced in patients with ypN+ compared to ypN0 (Figure S3, Supplementary File). Patients with a ypN+ status and harboring the Cys326 allele experienced a hazard ratio of 8.86 (95% confidence interval 3.37–23.34, *p* = 1 × 10^−5^) to encounter a CSS event compared with individuals with ypN0 and homozygous for the Ser326 allele. Taking the subgroup analyses together, the relevance of the Ser326Cys polymorphism came particularly into effect in patients who were treated with 5-fluorouracil as a single agent chemotherapy concomitant to irradiation and who had a ypN+ status at the time of surgical resection.

### 2.8. Functional Assessment of OGG1 rs1052133

Baseline micronuclei rate was not affected by rs1052133. However, an increase with a statistical trend was noticed for the variant allele when carriers had received about 50% of the radiation dose. This association even came up a bit stronger when assessed at the end of the N-RCT (see Supplementary File, Figure S4). The variant allele associated with worsened clinical outcome was linked to a slightly enhanced genotoxicity in the N-RCT course, as determined by the micronuclei in the peripheral leucocytes. Noteworthy, when patient leucocytes (obtained prior to the N-RCT start) were irradiated ex vivo with a single fraction of 3 Gy, micronuclei formation was not affected by OGG1 rs1052133. In addition to the micronuclei, also nuclear plasma bridges as a result of genotoxic treatment were considered; however, no effect of rs1052133 was observed.

In a panel of 185 lymphoblastoid cell lines (LCLs) of Caucasian origin, cellular radiosensitivity was assessed in relation to the OGG1 rs1052133 polymorphism. This analysis revealed increased radioresistance in cells harboring the variant allele at rs1052133 (Supplementary File, Figure S5).

## 3. Discussion

This study has discovered a strong impact of the germ-line polymorphism rs1052133 in the OGG1 gene on clinical outcome. The strongest effect was seen for CSS (see Table 3), which was affected by this polymorphism to a similar extent than the established clinical feature of a positive nodal status upon N-RCT [23]. Affecting one out of six patients, pathological complete response was linked to substantially decreased hazard ratios, in particular in terms of OS. As expected, age was a prognosticator for OS, but not for the other outcome parameters. VMAT exhibited a trend for better local control compared with 3D radiotherapy. Notably, tumor staging and grading features did not exhibit any effect on the outcome of the patients nor did addition of oxaliplatin to N-RCT. This is in line with the literature data not supporting routine administration of oxaliplatin within N-RCT [24]. There are, however, controversial data arguing for use of oxaliplatin in this setting [25]. Interestingly, subgroup analyses of our data set suggest that OGG1 Cys326 carriers might benefit from additional oxaliplatin concomitant to irradiation and 5-fluorouracil. Application of adjuvant chemotherapy did not translate into any significant outcome benefit in the entire patient cohort, consistent with a systematic review and meta-analysis [26].

In the multivariable analyses, the strong impact of the OGG1 rs1052133 polymorphism on outcome was retained (Table 4). This indicates the effect of this polymorphism being independent to a large extent from the considered histopathological features concerning prognosis. Combining to a multiparametric score revealed particularly distinctions on CSS and OS (Figure 4). This argues for inclusion of this OGG1 polymorphism on prognostic outcome in addition to the already established features of pCR and ypN status. This might be of particular clinical relevance as current attempts seek for organ preservation in selected patients with rectal cancer upon radio(chemo)therapy followed by consolidation chemotherapy.

Unlike with clinical outcome, none of the eight presented genetic polymorphisms affected initial tumor staging parameters or histopathologically assessed tumor response upon N-RCT when associations were adjusted for multiple testing.

Oxidative DNA damage is considered a major mechanism by which ionizing radiation exerts cytotoxicity. Formation of 8-hydroxydeoxyguanosine (8-OH-dG) occurs upon radiation exposure and is repairable in a timely manner [27]. Presence of non-repaired 8-OH-dG may cause a false incorporation of an adenine, resulting in a transversion mutation [28]. In human cells, 8-oxoguanine DNA glycosylase (OGG1) is crucially involved in removal of 8-OH-dG [29,30].

The OGG1 rs1052133 variant allele, which encodes for an amino acid substitution resulting in an exchange of Ser326 to Cys326, seems to confer a reduced function to the OGG1 enzyme [31]. Mutagenesis due to 8-OH-dG was less efficiently inhibited by the Cys326 variant [32]. The Cys variant seems to be more prone toward oxidative stress [33].

In addition, it was demonstrated by immunofluorescence that proper dynamic relocalization of the human OGG1 protein is vigorously disturbed in presence of Cys326 due to an altered phosphorylation status. Specifically, though transported into the nucleus, the mutant protein seems to be excluded from the nucleolus during the S-phase of the cell cycle [34]. This might be of particular relevance as the ribosomal DNA of the nucleoli is very sensitive to ionizing radiation [35]. Furthermore, in the Cys326 mutant protein, the associations of OGG1 with soluble chromatin and with the nuclear matrix during interphase as well as with condensed chromatin during mitosis are also disrupted [34]. These dramatic changes might render the mutant less effective for base excision repair. In mice, the peak activity following ROS exposure was 12 h later for the Cys326 in comparison with the Ser326 variant [21]. Similarly, upon pro-oxidant treatment, OGG1-initiated base excision repair was found to be transiently weaker for the Cys326 allele, possibly due to cysteine residue oxidation [36].

A functionally mitigated action of OGG1 in presence of Cys326 is supported by our micronuclei assessment with an increasing association during the course of N-RCT. The moderate effect we have observed is very nicely in line with that reported by Sinitsky et al. [37]. As in our investigation, they found a slightly enhanced micronuclei formation in presence of the Cys326 variant, with no impact on nuclear plasma bridges or the nuclear division index.

Though ex vivo irradiation of peripheral leucocytes induced micronuclei formation in our study in a highly statistically significant manner, no alteration by Cys326 was seen. This suggests that the Cys326 effect rather becomes evident in primordial lymphogenic precursor cells, residing in the bone marrow or in lymphatic tissues, than in the highly differentiated peripheral lymphocytes. The complex molecular cascades following the action of OGG1, finally resulting in micronuclei formation, may differ between the type and differentiation status of cells.

In fact, as demonstrated by the assessment of cell viability performed on highly proliferating LCLs, the Cys326 allele was related to enhanced radioresistance. This observation with viability as a cellular endpoint may provide a plausible link to the worse CSS and OS seen in patients with this variant. Based on the mitigated function of the OGG1 Cys326 variant, cells exposed to ROS stress may accumulate cellular damages. This, in turn, may increase the mutational burden as supported by the abovementioned reports with respect to carcinogenesis [32,33]. Unlike non-malignant cells, tumor cells may better overcome the OGG1 Cys326 malfunction by upregulation of other components involved in the orchestra of antioxidative and DNA repair functions. Possibly, such adaptation processes might even represent an over-compensation by which such cells acquire increased resistance towards ROS stress. By that, cells with OGG1 Cys326 may be less prone to cytotoxicity of irradiation as seen for LCLs. As similar effects are hypothesized for tumor cells, the link to a worse CSS as a result of enhanced treatment resistance appears at least conceivable.

The detrimental effect of the OGG1 Cys326 variant on clinical outcome may be reversed by addition of oxaliplatin to the N-RCT setting. Interestingly, as cytotoxicity of oxaliplatin was found to be in part unrelated to oxidative DNA damage [38], tumor cells might be prone towards this agent despite a possibly enhanced anti-oxidative capacity.

How is it conceivable that a variant with mitigated DNA repair capacity confers a worse outcome, in particular in terms of CSS? Is it rather the course of the disease or treatment-related? This could not sufficiently be answered in absence of a control cohort without medical intervention. One might argue that sensitivity toward a genotoxic regimen should be augmented in case of diminished DNA repair capacity. If that is the issue, Cys326 carriers should rather have faced a better than worse outcome. However, Cys326 might be a feature of more aggressive tumors. The homozygous *GG* genotype at rs1052133 was more frequently present initially in N2-N3 situations, but there were only few such cases in our study. In addition, this *G* allele showed a trend for over-representation in diseases, which remained ypN+ upon neoadjuvant therapy. As demonstrated by subgroup analyses, the presence of Cys326 was particularly detrimental in patients with ypN+ and treated with single 5-FU agent chemotherapy concurrent to irradiation (Figure S1, Supplementary File). In other words, carriage of Cys326 might define a patient group that do not adequately respond to 5-FU. This might plausibly translate, at least in part, in an enhanced rate of ypN+, for which the negative effect of Cys326 on outcome appeared particularly evident (Figure S3).

The assumption that the Cys326 effect is rather due to the biology of the disease than the treatment might be assumed in view of several reports on carcinogenesis. The risk for urinary bladder cancer in Japanese and for gallbladder cancer in Chinese and Indian population was substantially increased for carriers of the Cys326 variant [39,40,41]. However, respective data for other cancer entities are equivocal, such as lung carcinoma [42,43,44] or head and neck cancers [45,46]. In meta-analyses addressing the impact of the Ser326Cys polymorphism on colorectal cancer risk, no association was observed in one study [47], whilst two others reported Cys326 to be a risk allele in Caucasians [48,49]. More specifically, OGG1 Cys326 has been related to an increased cancer risk in the rectum but not at other sites in the colon [50]. However, if a person already is at enhanced risk for colorectal cancer due to deficiencies in DNA mismatch repair causing Lynch syndrome, this risk was not further elevated by low penetrance alleles such as the OGG1 Cys326 variant [51]. Finally, when considering the entire body of available literature, this variant might be attributed a small contribution to (colo)rectal cancer risk. If so, our observations might, at least in part, reflect the tumor biology and thereby the natural course of the disease.

Nevertheless, an interaction of this genetic variant with the treatment regimen is still possible. Interestingly, as sub-group analyses clearly demonstrated, the effect of rs1052133 on CSS is restricted to the patients treated with 5-FU as a single chemotherapeutic agent during N-RCT. Furthermore, it remains to be assessed whether this genetic polymorphism discriminates outcome also under new regimen such as RAPIDO for locally advanced high-risk rectal cancer [8].

Determination of the OGG1 Cys326 variant might add a benefit in two instances: According to the aforementioned subgroup analysis, carriers of the Cys326 variant might profit from an intensified chemotherapy component within the N-RCT course, i.e., by adding oxaliplatin. Moreover, this OGG1 polymorphism might, given its strong discrimination of CSS and OS together with the histopathological features of pCR and ypN status, assist in the adjuvant setting to guide further treatment and intensity of aftercare.

The here presented study has some limitations. Though the patients were treated according to well-defined protocols, there were some inherent treatment particularities. Whereas escalation of chemotherapy by adding on oxaliplatin to the N-RCT and technical evolutions of radiotherapy were well documented and a relevant bias could be ruled out, this is more difficult with regard to further adjuvant chemotherapy after surgery. The latter was intended and administered to the majority of patients; however, substantial heterogeneity occurred in the dosages and completion rates of the adjuvant chemotherapy. We were not able to fully control for these uncertainties as these treatments were all conducted outside our department. 

Though the patients were accrued in a prospective fashion according to established study protocols, the genetic analyses have to be considered as retrospective and thus hypothesis-generating, not hypothesis-proving. With OS and CSS regarded as the most relevant outcome parameters, the relatively low event rate even over a long time frame renders the analysis susceptible to a limited number of cases driving the statistical significance. Thus, further investigations, at best in a prospective clinical fashion, are necessary to confirm whether the OGG1 rs1052133 polymorphism is a relevant prognostic biomarker in locally advanced rectal cancer treated in a multimodal fashion. Future or ongoing trials on rectal cancer might assess the impact of this genetic marker.

## 4. Patients and Methods

### 4.1. Eligibility Criteria

Patients treated in a multimodal setting with N-RCT at the Göttingen Medical University Center, a tertiary medical institution, between 01/1998 and 08/2016, for locally advanced cancer in the middle or lower third of the rectum without distant metastases, were considered for this study. Treatment was according to the study protocol (see below). Out of the initial number of 388 patients, 101 were excluded from this study for several reasons. Patients with conditions that might strongly bias the clinical outcome upon multimodal therapy were removed from further analysis, i.e., initial presence of distant metastasis (*n* = 30) or a synchronous second malignancy (*n* = 4). A minimum follow-up of three months was defined to assess the effects of N-RCT on survival. Thus, patients who had a shorter follow-up (*n* = 3) or who died within three months post-surgery (*n* = 3) were also deemed not eligible. This is reasoned by the fact that some patients already might have hidden distant metastasis that was undetected at initial staging but became prevalent within a short timeline upon surgery. Therapy-related causes for exclusion comprise changes in the therapy concept (*n* = 4), irradiation applied at another institution with no data available (*n* = 2), or stopping the N-RCT before reaching 80% of the intended radiation dose (*n* = 12). For a relatively high number of patients, no DNA was available for genotyping, resulting in a further drop-out of 43 patients. Finally, 287 patients were eligible for the genotype–outcome analyses (see flowchart in Figure 1 above).

### 4.2. Disease Staging

Initial staging examinations included medical history, clinical examination, full blood count, blood concentration of carcinoembryonic antigen, abdominal ultrasound, chest X-rays, and pelvic MRT or contrast-embedded CT of the pelvis. Biopsies were taken during rigid rectoscopy with endoscopic ultrasound. The distribution of tumor stages is shown above in Table 2. Overall, 60 patients were classified as AJCC stage II (20.9%) and 227 as AJCC stage III (79.1%) before treatment (according to AJCC 8th edition, 2017). The lower border of all tumors was between 0 and 12 cm measured from the anal verge. All tumors were histologically determined as adenocarcinoma.

### 4.3. Multimodal Treatment Sequence

Treatment regimens consisted of N-RCT, TME surgery, and further adjuvant chemotherapy, as per the study protocol or depending on postsurgical risk factors. Treatment was conducted according to the protocols of four trials that differed essentially by two different chemotherapy strategies applied in parallel to radiotherapy, and, if indicated, also in an adjuvant setting. 5-fluorouracil as a single chemotherapeutic agent was administered in the experimental arm of the phase III CAO/ARO/AIO-94 trial [7], the standard arm of the phase III CAO/ARO/AIO-04 trial [52], and the single arm biomarker TransValid-A (NCT03034473) study. Oxaliplatin in conjunction with 5-fluorouracil was added in the experimental arm of the phase III CAO/ARO/AIO-04 trial and in the phase I/II TransValid-B (EudraCT 2011-004228-37) trial.

Radiotherapy was delivered using a linear accelerator. The intended total dose was 50.4 Gy with a daily fraction of 1.8 Gy five times per week. Given the long treatment period, radiation techniques applied for patients of this study evolved from 3D-planned over intensity-modulated (IMRT) to volumetric-modulated arc radiotherapy (VMAT). Prescribed radiation dosing was in accordance with each the current version of the International Commission on Radiation Units and Measurement Report.

Concomitant to radiotherapy, all patients received chemotherapy of which the full planned dose was achieved in 95% of the subjects (Table 2). Almost two thirds (184/287) obtained 5-fluorouracil (5-FU) as the sole agent within the course of N-RCT. This substance was administered at 1000 mg/m^2^/d as a continuous infusion for 120 h during the first and fifth week of N-RCT.

The other third of patients (103/287) was treated by an intensified regimen comprising a combination of 5-FU with oxaliplatin. In this case, the dose density of 5-FU was reduced to 250 mg/m^2^ TBSA/d supplied by four 7-day pumps from d1 to d14 and d22 to d35. Oxaliplatin (50 mg/m^2^ TBSA) was applied four times during N-RCT at d1, d8, d22, and d29 over a 2 h-infusion prior to plugging the 5-FU pump. For patients treated according to the TransValid-B protocol (*n* = 36), the N-RCT was followed by three additional cycles of FOLFOX (folinic acid, 5-FU, oxaliplatin) prior to rectal resection with total mesorectal excision (TME) performed six weeks after completion of N-RCT or three weeks subsequent to the 3rd FOLFOX cycle in TransValid-B.

The majority of patients (214/287, 75%) was assigned to further adjuvant chemotherapy. Treatment start was scheduled one month after surgery. Of those, 147 received 5-FU (bolus 500 mg/m^2^ TBSA/d) as monotherapy given in four five-day cycles every five weeks. Eight of these patients did not obtain the full adjuvant 5-FU dose. One patient got the 5-FU prodrug capecitabin. Intensified adjuvant chemotherapy was delivered according to the experimental arm of the CAO/ARO/AIO-04 trial. One month after surgery, 100 mg/m^2^ TBSA oxaliplatin, 400 mg/m^2^ TBSA folinic acid, both in a two-hour infusion, and 5-FU 2400 mg/m^2^ TBSA in a 46 h continuous infusion were given every two weeks up to 8 cycles total. Out of 66 patients assigned to this treatment, 42 completed eight cycles of the adjuvant chemotherapy as planned. Patients treated within the TransValid-B trial received no further treatment as they already got three additional chemotherapy cycles between the N-RCT and surgery.

### 4.4. Assessment of Treatment Outcome

Patients were followed-up until March 2020. As the outcome parameters, the freedom from local regional relapse, freedom from distant metastasis, cancer-specific survival, and overall survival, abbreviated as FLR, FDM, CSS, and OS, were assessed.

### 4.5. Genotyping

Eight genetic polymorphisms in seven genes, which code for enzymes involved in handling reactive oxygen species, were genotyped. These polymorphisms were selected based on the literature as having medical or functional relevance (see column “Reference” in Table 1). The selected sites were genotyped by the primer extension method (SNaPshot™, Applied Biosystems, Foster City, CA, USA) in DNA isolated from the peripheral blood cells of the patients. Hardy–Weinberg equilibrium was fulfilled for all eight genotyped sites (all *p*-values > 0.4 according to Pearson’s χ^2^-test). Sequences of the primers for multiplex PCR and of the primers for extension at the assayed polymorphic site (“SNaPshot primers”) are provided in Table S1, in the online Supplementary File.

### 4.6. Micronuclei Assessment

In a subset (*n* = 148) of the considered clinical cohort, peripheral blood samples were drawn prior to, in the middle, and at the end of the N-RCT. Micronuclei formation was assessed as a hallmark for treatment-conferred genotoxicity, as described previously [53,54]. Briefly, using patient blood lymphocytes, the cytokinesis-block micronucleus cytome assay was performed. The blood lymphocytes were obtained by density-gradient centrifugation. Phytohaemagglutinin was used to stimulate the cells and further cell division was stopped via cytochalasin B. Finally, upon cytospin centrifugation, the cytogenetic damage (micronuclei and nucleoplasmatic bridges formation) was quantified.

### 4.7. Cell viability Assessment

Lymphoblastoid cell lines (LCLs) were used as a model for highly proliferating cells and were purchased from the Coriell institute (http://ccr.coriell.org, accessed on 3 June 2021) and are listed in Table S2. Radiosensitivity of 185 LCLs of Caucasian origin was assessed as cellular viability in response to a single fraction of 3 Gy photons. Irradiation was carried out by a RS 225 X-ray cabinet (Gulmay Medical Systems, Camberley, Surrey, UK) using 200 kV, 15 mA, and filtration with 0.5 mm copper. Irradiated and non-irradiated LCLs, which served as the controls, were incubated for 24 h in an incubator at 37 °C and with 5% CO_2_. Then, alamarBlue^TM^ reagent (Thermo Fisher Scientific, Waltham, MA, USA) was added. Upon a further 4 h of incubation, the cell viability was recorded by a microplate reader (Tecan Ultra, Männedorf, Switzerland). For each LCL, cell viability of an irradiated sample was ascertained as the percentage in relation to the non-irradiated control. Genotypes at OGG1 rs1052133 for the individual LCLs were obtained from the 1000 Genomes Project (https://www.internationalgenome.org, accessed on 3 June 2021).

### 4.8. Ethical Approval

The study procedures were conducted in accordance with the ethical standards on human experimentation of the World Medical Association Declaration of Helsinki. Approval was obtained by the Ethics Committee of the University of Göttingen, Germany (application numbers 20/9/95, 9/8/08). All participating patients or their legal representatives have given written informed consent.

### 4.9. Statistical Analyses

The impact of the genetic and non-genetic features on the treatment outcome parameters was evaluated by Kaplan–Meier plots along with a log-rank test and by univariable and multivariable Cox regression. A nominal level of statistical significance was set at *p* < 0.05. Considering multiplicity testing of the genetic data, which were evaluated in relation to clinical outcome in an exploratory way, the respective *p* values were adjusted for the numbers of genetic polymorphisms. Data were analyzed using the software SPSS (v 26, IBM) and R 4.0.2 with the “KMWin” (Kaplan–Meier for Windows) plugin [55].

## 5. Conclusions

We report on a germline genetic polymorphism in OGG1 resulting in the missense mutation Ser326Cys, strongly affecting outcome upon multimodal treatment of rectal cancer. Carriers of the Cys variant experienced worse outcome. This finding attracts relevance for several reasons. First, 37% of the general population harbor at least one variant allele of this polymorphism. Second, a consistent effect of this genetic marker on the reported outcome parameters was observed, having the strongest relation to cancer-specific survival and even holding statistical significance upon adjustment for multiple testing. Third, in the multivariable analyses, the effect of this biomarker on CSS and OS surpassed those of pCR and ypN status, two established factors with high prognostic significance. A particularly strong discrimination of CSS and OS outcome could be achieved with a multiparametric score combing OGG1 rs1052133, pCR, and ypN status. Fourth, one might speculate on the differential mechanistic actions of the two alleles at amino acid position 326 in OGG1. In line with mitigated base excision repair reported in the literature for Cys326, we found an increased rate of micronuclei formation upon irradiation, suggestive of reduced clearance from ROS-driven DNA damage. The Cys326 variant might be linked to enhanced radioresistance as proposed by data obtained in a panel of 185 LCLs. Further clinical and functional studies are intended to define the medical conditions in which this biomarker may make a prognostic difference and should thus be considered as a target for future therapeutic approaches.

## Figures and Tables

**Table 1:** Characteristics of the genotyped polymorphisms.

Gene	SNP	Genomic Localization ^1^	Affected Element ^2^	Alleles ^3^	MAF ^4^	References
CAT	rs1001179	11:34438684	upstream, −250 bp	*C > T*	0.216	[13,14]
	rs769214	11:34438170	upstream, −764 bp	*A > G*	0.346	[15,16]
CYBA	rs1049255	16:88643329	3′-UTR	*A > G*	0.495	[17]
GPX1	rs1050450	3:49357401	coding, missense	*C > T*	0.239	[18,19]
MPO	rs2333227	17:58281401	upstream, −466 bp	*G > A*	0.192	[10,20]
OGG1	rs1052133	3:9757089	coding, missense	*C > G*	0.203	[21,22]
SOD2	rs4880	6:159692840	coding, missense	*T > C*	0.486	[10,12,14,19]
SOD3	rs699473	4:24795181	upstream, −297 bp	T > C	0.356	[14]

^1^ According to the genome build GRCh38.p12 (https://www.ncbi.nlm.nih.gov/, accessed on 30 November 2020), with the chromosome number and the chromosomal position given. ^2^ In case the polymorphic site is located upstream of the transcribed gene the distance to the *ATG* start codon of the major transcript (as annotated in GRCh38.p12) is indicated in basepairs (bp). ^3^ The denoted alleles refer to the sense strand of the respective gene. ^4^ MAF = minor allele frequency, i.e., the frequency right to the “>” symbol in the “Allele” column, as observed in the here reported study.

**Table 2:** Baseline patient, disease, and treatment characteristics (based on the 287 eligible patients). If not otherwise stated, per item the respective numbers with percentage in brackets are denoted.

Item	Numbers (%)
**Age (years):** median (min–max)	64.4 (20.8–85.4)
**Female**	95 (33.1)
**T category ^1^**	
T1	0 (0)
T2	6 (2.1)
T3	256 (89.2)
T4	25 (8.7)
**Nodal status ^1^**	
N0	60 (20.9)
N+	227 (79.1)
**AJCC stage ^2^**	
II	60 (20.9)
III	227 (79.1)
**Radiotherapy ^3^**	
Completed as planned	282 (98.3)
80% ≤ dose < 100%	5 (1.7)
**Type of chemotherapy within** N**-RCT**	
5-FU mono	184 (64.1)
5-FU + oxaliplatin ^4^	103 (35.9)
**Chemotherapy within** N**-RCT completed ^5^**	273 (95.1)
**Pathological complete response**	48 (16.7)
**T category upon** N**-RCT**	
ypT0	52 (18.1)
ypT1	32 (11.2)
ypT2	71 (24.7)
ypT3	123 (42.9)
ypT4	9 (3.1)
N **category upon** N**-RCT**	
ypN0	203 (70.7)
ypN1	61 (21.3)
ypN2	23 (8.0)
**Postoperative chemotherapy**	214 (74.6)

^1^ Determined by imaging (CT, MRT, and/or endorectal ultrasound). ^2^ American Joint Committee on Cancer (AJCC), TNM Staging System for rectal cancer 8th edition, 2017. ^3^ Planned radiation dose was 50.4 Gy in 28 × 1.8 Gy fractions, 5 times/week. Only patients who received at least 80% of the initially prescribed dose were included, i.e., patients with earlier stop of radiotherapy were excluded (*n* = 12, flowchart Figure 1). ^4^ This comprises 67 patients treated within or analogous to the experimental arm of the CAO/ARO/AIO-04 trial (with two having received the prodrug capecitabine instead of 5-FU) and 36 patients with treatment according to the TransValid-B protocol. ^5^ In 14 patients, the chemotherapy administered was prematurely stopped due to intolerable side effects.

**Table 3:** Univariable Cox regression for assessing the impact of patient baseline, tumor, treatment, and OGG1 genotype on freedom from locoregional relapse (FLR), freedom from distant metastasis (FDM), cancer-specific survival (CSS), and overall survival (OS). Nominal *p* values < 0.05 are highlighted in bold.

Variable	FLR		FDM		CSS		OS	
	Hazard Ratio (95% CI)	*p* Value	Hazard Ratio (95% CI)	*p* Value	Hazard Ratio (95% CI)	*p* Value	Hazard Ratio (95% CI)	*p* Value
**Age** (per year)	0.98 (0.93–1.03)	0.36	1.00 (0.98–1.03)	0.96	1.01 (0.97–1.04)	0.78	1.03 (1.00–1.06)	**0.02**
**Sex** Female (95) vs. male (192)	1.12 (0.38–3.28)	0.84	0.76 (0.42–1.38)	0.37	1.32 (0.63–2.78)	0.46	1.07 (0.60–1.91)	0.81
**T category** T4 (25) vs. T2-T3 (262)	0.70 (0.09–5.34)	0.73	0.35 (0.09–1.44)	0.15	0.67 (0.16–2.83)	0.59	0.96 (0.38–2.42)	0.94
**N category** N+ (227) vs. N0 (60)	1.23 (0.49–3.05)	0.66	0.74 (0.46–1.22)	0.24	0.70 (0.35–1.39)	0.31	0.74 (0.44–1.23)	0.24
**Radiation technique ^1^** VMAT (119) vs. 3D-RT (157)	0.24 (0.05–1.07)	0.06	0.89 (0.52–1.53)	0.67	0.82 (0.38–1.77)	0.61	0.62 (0.33–1.15)	0.13
**N-RCT with oxaliplatin** Yes (103) vs. no (184)	0.66 (0.21–2.08)	0.48	0.78 (0.44–1.37)	0.38	1.06 (0.50–2.22)	0.88	1.39 (0.81–2.38)	0.23
**pCR** Yes (48) vs. no (239)	0.69 (0.16–3.04)	0.62	0.26 (0.81–0.83)	**0.02**	0.16 (0.02–1.18)	0.07	0.09 (0.01–0.64)	**0.02**
**ypN+** Yes (84) vs. no (203)	1.85 (0.66–5.19)	0.25	3.09 (1.83–5.23)	**<0.001**	3.47 (1.69–7.15)	**<0.001**	2.48 (1.46–4.24)	**<0.001**
**Adjuvant chemotherapy** Yes (214) vs. no (73)	1.17 (0.33–4.17	0.81	0.99 (0.53–1.85)	0.98	0.72 (0.31–1.63)	0.42	0.66 (0.36–1.21)	0.18
**OGG1 rs1052133** *GG* + *GC* (105) vs. *CC* (182)	2.77 (0.99–7.79)	0.05	1.72 (1.02–2.91)	**0.04**	3.64 (1.70–7.78)	**<0.001**	2.12 (1.24–3.63)	**0.006**

^1^ For assignment of a patient to the radiation technique, it was necessary that at least 80% of the fractions were administered as either volumetric-modulated arc therapy (VMAT) or three-dimensional radiotherapy (3D-RT), respectively. Thus, eleven patients did not meet this criteria (i.e., not unequivocally attributable as substantial fractions of both techniques applied).

**Table 4:** Multivariable Cox regression with the same outcome parameters as in Table 3. Variables that had a *p* < 0.05 with any of the outcome measures in the univariable analysis were put in the models (in bold). ^2^pCR = pathological complete response at time of surgical resection. ^3^ypN+ = viable tumor cells in lymph nodes at surgical resection.

Variable	FLR		FDM		CSS		OS	
	Hazard Ratio (95% CI)	*p* Value	Hazard Ratio (95% CI)	*p* Value	Hazard Ratio (95% CI)	*p* Value	Hazard Ratio (95% CI)	*p* Value
**Age** (per year)	0.98 (0.94–1.03)	0.43	1.00 (0.98–1.03)	0.88	1.01 (0.98–1.04)	0.67	1.03 (1.01–1.06)	**0.02**
**pCR ^2^** Yes (48) vs. no (239)	0.76 (0.16–3.69)	0.73	0.37 (0.11–1.24)	0.11	0.22 (0.03–1.73)	0.15	0.11 (0.01–0.77)	**0.03**
**ypN+ ^3^** Yes (84) vs. no (203)	1.58 (0.53–4.76)	0.41	2.51 (1.46–4.32)	**<0.001**	2.41 (1.14–5.07)	**0.02**	1.71 (0.99–2.94)	0.06
**OGG1 rs1052133** *GG* + *GC* (105) vs. *CC* (182)	2.63 (0.92–7.48)	0.07	1.64 (0.97–2.78)	0.07	3.41 (1.58–7.34)	**0.002**	2.11 (1.23–3.62)	**0.007**

## Data Availability

Primer sequences are provided in the Supplementary Materials. The datasets used for this manuscript, anonymous for the individual patients, could be obtained from the corresponding author by reasonable request.

## Supplementary Materials

The following are available online at https://www.mdpi.com/article/10.3390/cancers13112805/s1, Figure S1: CSS in dependence on the OGG1 rs1052133 polymorphism stratified by sort of chemotherapy concomitant to irradiation. Figure S2: CSS in dependence on the OGG1 rs1052133 polymorphism stratified by whether adjuvant chemotherapy was given or not. Figure S3: CSS in dependence on the OGG1 rs1052133 polymorphism stratified by post-surgery ypN status. Figure S4: Micronuclei rate in patient leucocytes during the time course of N-RCT stratified by OGG1 rs1052133. Figure S5: Radiosensitivity of lymphoblastoid cell lines in dependence on OGG1 rs1052133. Table S1: Primer sequences for multiplex PCR and polymorphic sites assayed. Table S2: List of employed lymphoblastoid cell lines.
