# Supplementary material for: 8-Oxoguanine DNA Glycosylase (OGG1) Cys326 Variant: Increased Risk for Worse Outcome of Patients with Locally Advanced Rectal Cancer after Multimodal Therapy
Source: Cancers (Basel). 2021 Jun 4;13(11):2805. doi: 10.3390/cancers13112805 (PMC8200071; doi:10.3390/cancers13112805)
Supplement: Supplementary file 1 [file cancers-13-02805-s001.zip › cancers-1206119-supplementary.pdf]

Supplementary Materials:

8-Oxoguanine DNA Glycosylase (OGG1) Cys326 Variant: Increased Risk for Worse Outcome of Patients with Locally Advanced Rectal Cancer after Multimodal Therapy

Table S1. Primer sequences for multiplex PCR (MPCR) and polymorphic sites to be assayed (SNaPshot™ primer).

Gene	SNP	MPCR primer, forward 5ˈ->3ˈ	MPCR primer, reverse 5ˈ->3ˈ	SNaPshot™ primer, 5ˈ->3ˈ
CAT	rs1001179	CCTCGGGAGGACTGCCTTCTGA	CCCCACCCAGCAGGGTCTAAGTATTC	$(ACTGACTG)_2$ACTCGCCCTGGGTTCGGCTAT
CAT	rs769214	TCCTAGCACCTGAGGAGGTGTAGAAATC	CTGCCAAATTGGCTTCTTTAAACACTG	CAGAAATCTGCTTCCCCAAATTTTAC
CYBA	rs1049255	GTGACCGACGAGGTCGTGTGA	GGAAGGCGATGCTGATGTTAATGAAAC	CCCGGACCTGCCCTCCC
GPX1	rs1050450	AGGGCAAAATCCCGGTGACTCATAG	TGGTCTGGCAGAGACTGGGATCAAC	$(TGAC)_4$TCCCTGCTGTCTCAAGGGC
MPO	rs2333227	CGCCCTAGCCTCTAGCCACATCATC	ACCAGAATTTCAGAGGGGTTAGTTGTGTG	GACTTGACCTCAAGTGATCCACC
OGG1	rs1052133	ACTCCACCCTCCTACAGGTGCTGTTC	GTGGGGATGGGGAGAGAGAAGTG	$(TGAC)_8$TCCGACCTGCGCCAAT
SOD2	rs4880	CGGGCTGTGCTTTCTCGTCTTC	ATCTGCGCGTTGATGTGAGGTTC	$(GACT)_2$GAGCCCAGATACCCCAAA
SOD3	rs699473	CACCCGCTGCTCTGCTCTCATAG	CGGCTTGTAGCCAGTGACCTTCAG	CTGACTGCACCTTGCAAATGTGC

Table S2. Employed lymphoblastoid cell lines with their identifier of the Coriell institute (http://ccr.coriell.org, accessed on 3 June 2021). These cell lines are all of Caucasian origin.

HG00096	HG00097	HG00099	HG00100	HG00101	HG00102	HG00103	HG00104
HG00106	HG00108	HG00109	HG00110	HG00111	HG00112	HG00113	HG00114
HG00116	HG00117	HG00118	HG00119	HG00120	HG00121	HG00122	HG00123
HG00124	HG00125	HG00126	HG00127	HG00128	HG00129	HG00130	HG00131
HG00133	HG00134	HG00135	HG00136	HG00137	HG00138	HG00139	HG00140
HG00141	HG00142	HG00143	HG00146	HG00148	HG00149	HG00150	HG00151
HG00152	HG00154	HG00155	HG00156	HG00158	HG00159	HG00160	HG00231
HG00232	HG00233	HG00234	HG00235	HG00236	HG00237	HG00238	HG00239
HG00240	HG00242	HG00243	HG00244	HG00245	HG00246	HG00247	HG00249
HG00250	HG00251	HG00252	HG00253	HG00254	HG00255	HG00256	HG00257
HG00258	HG00259	HG00260	HG00261	HG00262	HG00263	HG00264	HG00265
NA06984	NA06985	NA06986	NA06989	NA06993	NA06994	NA07000	NA07022
NA07034	NA07037	NA07051	NA07055	NA07056	NA07345	NA07357	NA11829
NA11831	NA11832	NA11839	NA11840	NA11843	NA11881	NA11882	NA11892
NA11893	NA11894	NA11919	NA11920	NA11830	NA11931	NA11932	NA11933
NA11992	NA11993	NA11994	NA11995	NA12003	NA12004	NA12005	NA12006
NA12043	NA12044	NA12045	NA12056	NA12057	NA12058	NA12144	NA12145
NA12146	NA12154	NA12155	NA12234	NA12239	NA12248	NA12249	NA12272
NA12273	NA12282	NA12283	NA12286	NA12287	NA12347	NA12348	NA12383
NA12399	NA12400	NA12413	NA12489	NA12546	NA12717	NA12718	NA12749
NA12750	NA12751	NA12761	NA12762	NA12763	NA12775	NA12777	NA12778
NA12812	NA12813	NA12814	NA12815	NA12827	NA12829	NA12830	NA12842
NA12843	NA12872	NA12873	NA12874	NA12875	NA12889	NA12890	NA12891
NA12892							

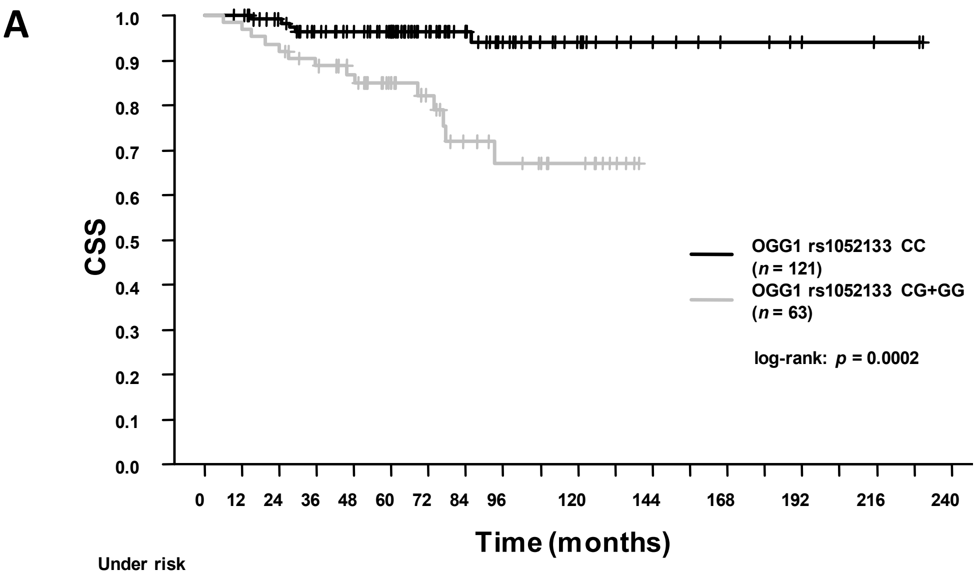

Under risk																				
OGG1 rs1052133 CC	120	110	99	92	79	55	42	31	23	18	13	10	8	6	6	3	3	2	2	0
OGG1 rs1052133 CG+GG	61	59	53	47	35	26	18	14	12	9	4	0	0	0	0	0	0	0	0	0

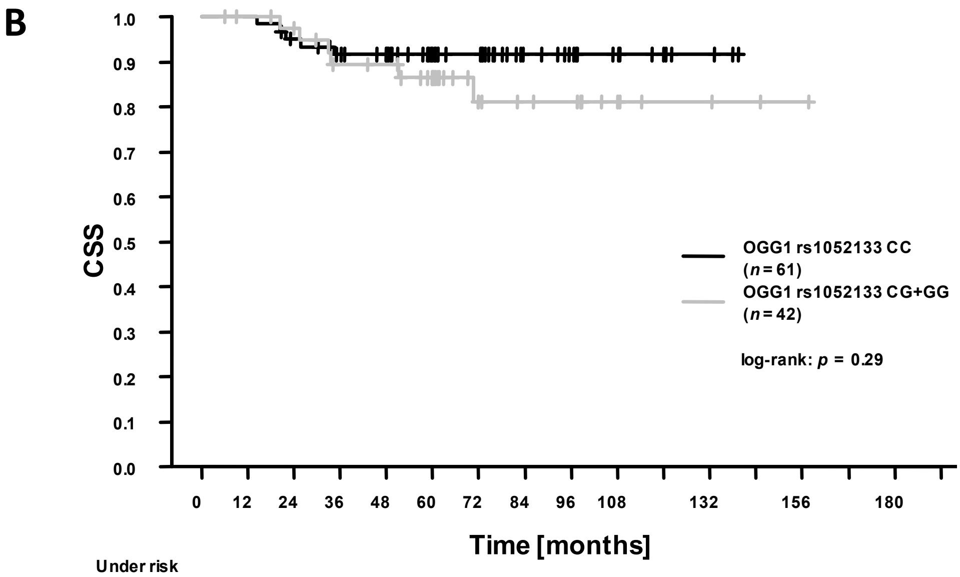

Under risk																
OGG1 rs1052133 CC	61	56	51	46	36	30	18	14	9	5	3	0	0	0	0	0
OGG1 rs1052133 CG+GG	40	38	32	31	24	13	11	10	6	3	3	2	1	0	0	0

Figure S1. CSS in dependence on the OGG1 rs1052133 polymorphism stratified by sort of chemotherapy concomitant to irradiation: 5-FU mono (panel **A**), addition of oxaliplatin (**B**).

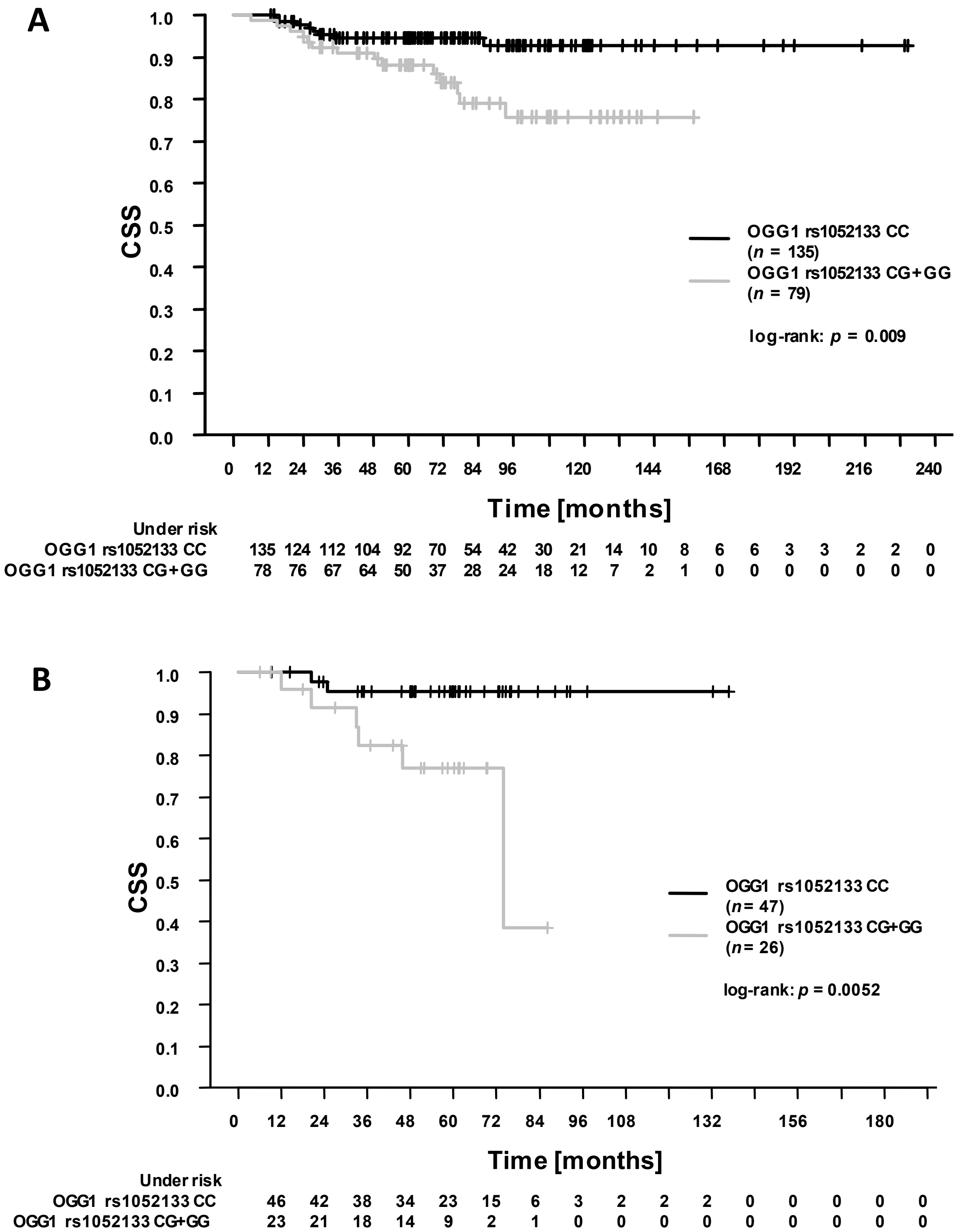

Figure S2. CSS in dependence on the OGG1 rs1052133 polymorphism stratified by whether adjuvant chemotherapy was given: yes (panel **A**), no (**B**).

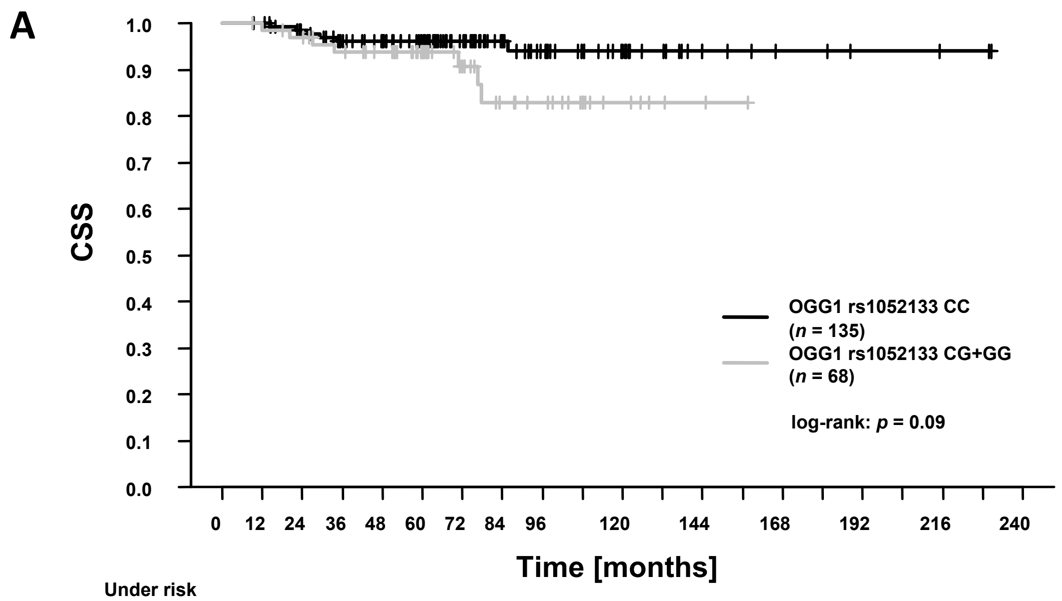

Under risk																				
OGG1 rs1052133 CC	134	125	114	106	88	66	47	36	27	20	15	9	7	5	5	3	3	2	2	0
OGG1 rs1052133 CG+GG	66	64	59	55	41	26	19	16	11	6	3	2	1	0	0	0	0	0	0	0

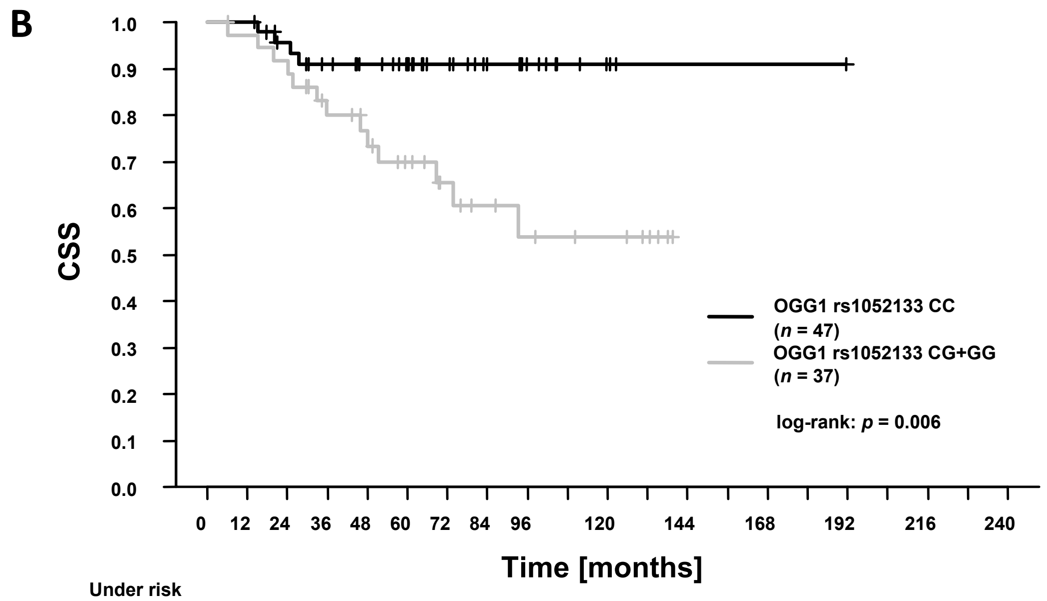

Under risk																				
OGG1 rs1052133 CC	47	41	36	32	27	19	13	9	5	3	1	1	1	1	1	0	0	0	0	0
OGG1 rs1052133 CG+GG	35	33	26	23	18	13	10	8	7	6	4	0	0	0	0	0	0	0	0	0

Figure S3. CSS in dependence on the OGG1 rs1052133 polymorphism stratified by post-surgery ypN status: ypN0 (panel **A**), ypN+ (**B**).

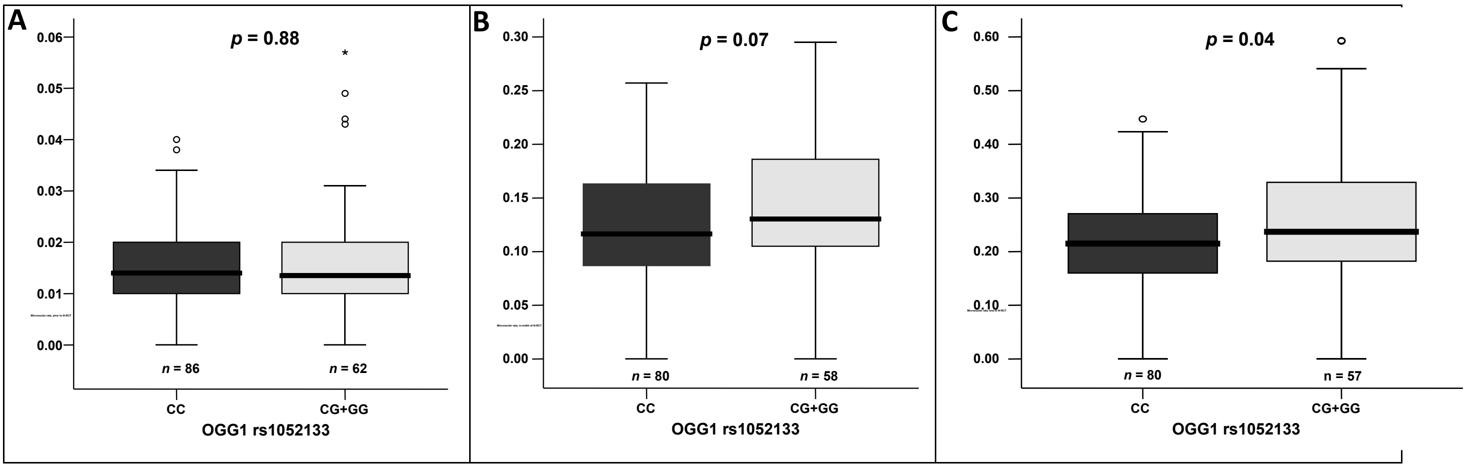

Figure S4. Micronuclei rate in patient leucocytes stratified by OGG1 rs1052133 during the time course of N-RCT: prior to start (panel **A**), in the midth (**B**), and at the end (**C**). The statistical difference was evaluated by Mann-Whitney U test with the respective P values given in the plots. Sample numbers for each group are given in the plots. The box-plots indicate the data distributions as follows: The rectangle represents 50% of the values of a given distribution with the lower horizontal line reflecting the 25%- (Q_{25}), and the upper the 75%-(Q_{75}) quartile. The difference of these two delimiters is called the interquartile distance (IQA). Values within 1.5-times of the IQA below Q_{25} or 1.5-times above Q_{75} are depicted by the whiskers of the blot (vertical line limited by short horizontal). Values out of this range are either marked by circles (>1.5-times, but ≤ 3-times of IQA referred to Q_{25} or Q_{75}) or asterisks (beyond 3-times of IQA on either side). Statistical assessment was performed by Mann-Whitney U test.

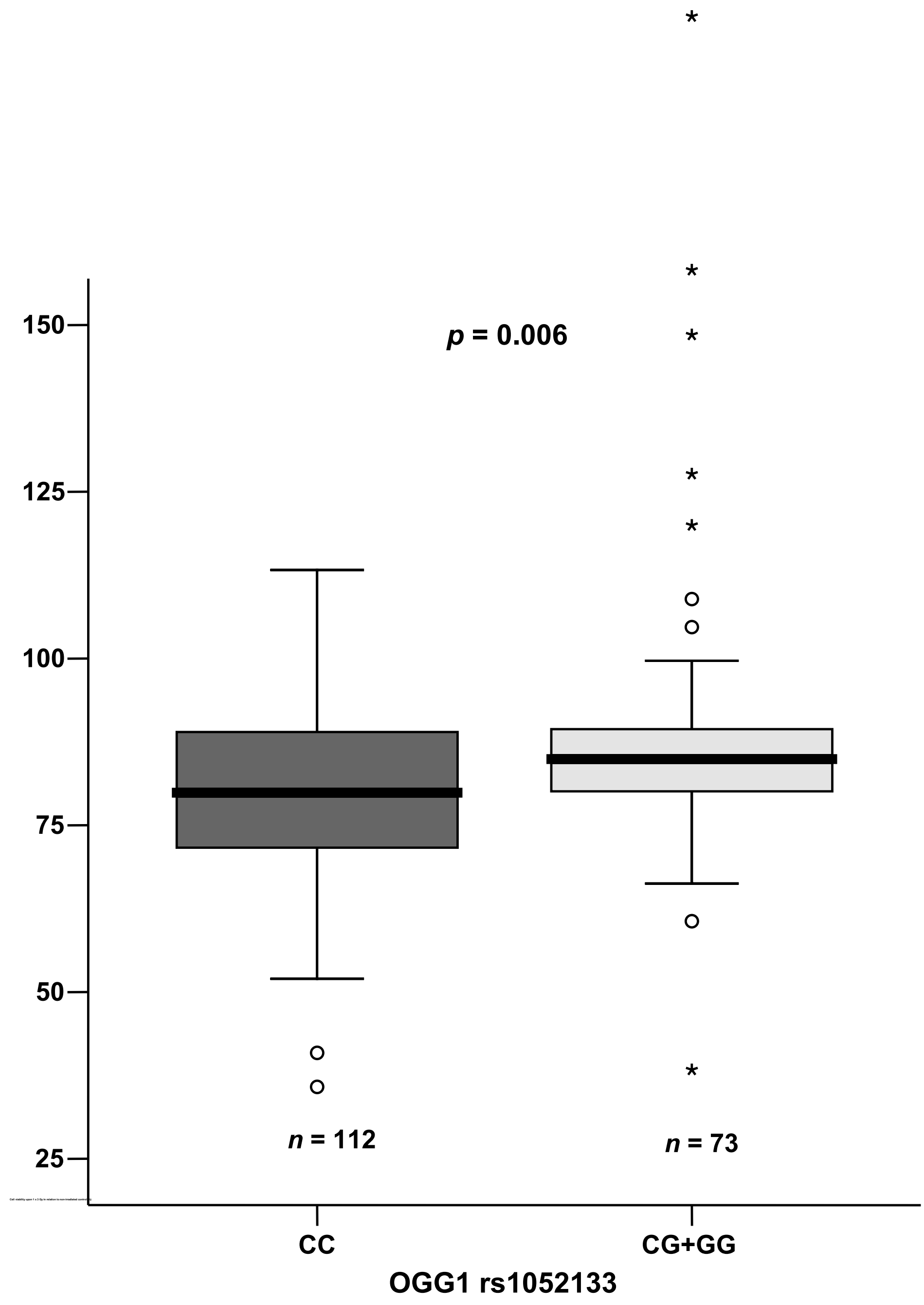

Figure S5. Cell viability of lymphoblastoid cells (LCLs) upon 30 h of 1 x 3 Gy photon irradiation in relation to non-irradiated control in dependence on the OGG1 rs1052133 status. Cell viability was determined using alamarBlue™ reagent. For box plot description and statistical assessment please refer to Figure S4.
